# The prevalence of online food delivery service usage and its association with anthropometric measurements in Muscat, Oman; a cross-sectional study

**DOI:** 10.1016/j.pmedr.2025.102966

**Published:** 2025-01-08

**Authors:** Ahmed Yahya Al Kharusi, Safa Khamis Ambusaidi, Marwa Abdullah Al Raisi, Haitham Mohammed Al Mahrouqi, Asma Ali Al Kendi, Muna Mohammed Almatrushi, Mohammed Juma Al Abdali, Sanjay Jaju, Maisa Hamed Al Kiyumi

**Affiliations:** a Department of Primary Care, Ministry of Health, Muscat, Oman; b Department of Primary Care, Armed Force Medical Services, Muscat, Oman; c Department of Family Medicine and Public Health, Sultan Qaboos University, Muscat, Oman; d Department of Family Medicine and Public Health, Sultan Qaboos University Hospital, University Medical City, Sultan Qaboos University,Muscat, Oman

**Keywords:** Online food delivery, Obesity prevalence, Anthropometrics, Socio-demographic factors, Cross-sectional study, Muscat-Oman

## Abstract

**Background:**

In Oman, the popularity of online food delivery services has soared since their introduction two years before the pandemic. This study aims to assess the prevalence of online food delivery service usage among Omani individuals in the Muscat region and explore its association with overweight/obesity.

**Methods:**

This is a cross-sectional study that was conducted in six randomly selected primary healthcare centres in the two most populous areas in Muscat region. Adults aged 18 years or older, who were able to read and write, and who attended the local health centre for any reason, were included. A consecutive sampling was used for enrolling subjects. A self-administered questionnaire was used and the study was conducted from January 2023 to June 2023. SPSS version 24 was used for statistical analysis.

**Results:**

A total of 467 participants were surveyed, revealing a high prevalence rate of online food delivery service usage (76.9 %,359). Younger age (*P* = 0.001), being single (*P* = 0.012), higher educational attainment (P = 0.001), absence of chronic diseases (*P* = 0.020),and physical inactivity (*P* = 0.028) were significant predictors. No association was found between online food delivery service usage and obesity/overweight (*P* = 0.109). While participants reported fair to good control over online food ordering, living alone emerged as a significant influencing factor, with dinner being the most commonly ordered meal.

**Conclusions:**

Our findings align with global trends, highlighting the convenience of online food services. However, the predominance of unhealthy food options raises concerns about the long-term health implications. Future research should include other regions of Oman and adopt prospective longitudinal designs.

## Highlights

• High prevalence (76.9 %) of online food delivery usage in Muscat, Oman, was identified.
• Usage significantly linked to younger age, single status, and higher education levels.
• Physical inactivity and living alone were associated with increased usage rates.
• No significant association between online food delivery usage and obesity/overweight.
• Findings underscore the need for research on long-term health impacts and regional trends.

## 1 Introduction

In recent years, the global demand for meal delivery services has increased significantly (Al-Busaidi et al., 2021). Online food delivery services (OFDS) are best suited to meeting such a huge demand. In Oman, OFDS were launched two years before COVID-19, but became more popular during the pandemic owing to the restrictions recommended for curbing the spread of the infection (Al-Busaidi et al., 2021). Various OFDS applications – such as Talabat, Akeed, Delivery Hero, Daleel 1010, etc. – gained popularity at the beginning of 2020. During the pandemic, a large number of people utilised these OFDS, considering them secure and convenient (Yousuf, 2020) Moreover, food service providers began encouraging their customers to use these online applications to order food, rather than going to the restaurant (Al-Busaidi et al., 2021). Many local Arabic and international restaurants, fast food providers (Kentucky Fried Chicken (KFC), McDonald's (McD), Pizza Hut, etc.), coffee shops and others offer online meal delivery services. A recent study from Kuwait reported a very high prevalence of usage of OFDS applications (87.6 %). Interestingly, fast food was the most regularly requested item, and around 76.4 % of people ordered it late at night (Almansour et al., 2020).

The OFDS market in other Gulf Corporation Countries, including Saudi Arabia, the United Arab of Emirates and Qatar, has grown significantly, with a market valuation estimated at $14.96 billion in 2024 (Statista, 2024a, Statista, 2024b). The surge in digital adoption has been driven by changing consumer behaviours, urbanisation, and increasing internet availability. In the United Arab of Emirates, adolescents and university students are notable users of OFDS applications, perceiving these services as convenient, yet expressing concerns about healthy food availability and food safety. Studies highlight that 70 % of adolescents prefer to order food online for its ease, but remain sceptical about the nutritional value of the meals offered (Saleh et al., 2024; Cheikh Ismail et al., 2024). Similarly, the adoption of OFDS in Saudi Arabia reflects cultural shifts toward technology integration, and a growing preference for fast and accessible dining options (Algheshairy et al., 2022).

According to the World Health Organization (WHO, 2024), adult obesity has more than doubled since 1990, while obesity among adolescents has increased fourfold. By 2022, approximately 2.5 billion adults aged 18 and older were classified as overweight, with 890 million of them obese.

Studies in Oman reveal a rise in the incidence of obesity and about 35.2 % of Omani people are obese, compared to 19 % of non-Omanis. In addition, a greater prevalence of obesity is found among females (40.9 %) in Oman than among males (28.2 %) (Ministry of Health and UNICEF Oman, 2018).

With the rapid increase in online fast-food consumption, concerns have been raised regarding the consequences of this habit and its link to the high prevalence rate of overweight and obesity (Stephens et al., 2020; Horta et al., 2022). Studies have associated OFDS use with weight gain and emotional eating (Jia et al., 2024). Additionally, the increased portion sizes, calorie content, and sodium levels in fast food over the past 30 years may have exacerbated the obesity epidemic through these platforms (Stephens et al., 2020). The Coronary Artery Risk Development in Young Adults (CARDIA) study, a large prospective longitudinal study of 3031 young people who were followed up for 15 years, revealed a twofold increase in insulin resistance among those who consumed fast food at least twice per week (Pereira et al., 2005).

Another observational study of university students in Indonesia found a significant association between ordering meals online and obesity [*p* < 0.001], but not with the type of food ordered [*p* = 0.99] (Harahap et al., 2020) Similarly, Kant et al. observed that people with more access to online food outlets had a greater likelihood of being overweight or obese (Kant et al., 2015).

A study done in the United Kingdom reported that the availability and abundance of OFDS was a factor that influenced their usage. Participants in areas with greater access to OFDS were more than twice as likely to order food online compared to those in less accessible areas (Keeble et al., 2021) A similar finding was corroborated by the study of Stephens et al. conducted in the USA. Similarly, a study conducted by Mokhtazar and Hamirudin in Malaysia revealed a strong relationship between the frequency of meal delivery service and waist circumference (Mokhtazar and Hamirudin, 2020).

According to a recent survey conducted in Oman, the majority of restaurant owners viewed OFDS favourably in terms of improved revenue and consumer trust (Al-Busaidi et al., 2021). To the best of our knowledge, this is the first study conducted in Oman that aims to assess the prevalence of OFDS usage, examine user attitudes in the Muscat region, and explore the relationship between online food consumption and obesity through BMI measurements.

## 2 Methodology

### 2.1 Study Design and Sampling Method

This is a cross-sectional study conducted from January 2023 to June 2023 in six randomly selected primary healthcare centres located in Al Seeb and Bausher, the two most densely populated areas in the Muscat region. Additionally, many people residing in the Muscat region originally come from other areas of Oman. Participants were recruited through consecutive sampling, where a well-trained triage nurse identified and invited eligible participants. Consecutive sampling was chosen for this study due to its practicality and efficiency in recruiting participants within the healthcare setting. Written informed consent was obtained from all participants, and those who declined participation had their reasons documented. Each participant was assigned a unique identifier and enrolled chronologically at each study site.

### 2.2 Inclusion and Exclusion Criteria

Inclusion criteria included Omani adults aged 18 years or older who were literate and attended the selected healthcare centres during the study period. Exclusion criteria included non-Omani individuals, those unable to read or write, patients who were critically ill and attending emergency services, and individuals who declined participation.

### 2.3 Survey Instrument

Data were collected using a structured, self-administered questionnaire divided into two sections. The first section captured socio-demographic details, including name, age, gender, educational level, employment status, income, medical history, and history of weight loss interventions. The second section assessed the usage of OFDS, focusing on patterns of use, frequency, influencing factors, most commonly ordered meals, and time of ordering.The questionnaire was adapted from previously validated studies in the literature (Almansour et al., 2020). It underwent forward and backward translation into Arabic, followed by a review by bilingual experts to confirm linguistic and conceptual equivalence. A pilot study was conducted with 20 participants from similar demographic backgrounds to refine the questionnaire for clarity and reliability. Feedback from the pilot study was incorporated into the final instrument.

### 2.4 Data Collection Procedures and Measures

Anthropometric measurements were collected under standardised conditions by trained staff nurses. Heights were measured using a stadiometer with participants standing upright without shoes, and weights were measured using a calibrated digital weighing scale. Measurements were recorded to the nearest 0.1 cm for height and 0.1 kg for weight. The classification of Body Mass Index (BMI) followed the World Health Organization (WHO) standards: underweight (<18.5 kg/m^2^), normal weight (18.5–24.9 kg/m^2^), overweight (25.0–29.9 kg/m^2^), and obese (≥30.0 kg/m^2^). A self-administered questionnaire was distributed after anthropometric measurements, ensuring privacy during data collection.

### 2.5 Sample Size Calculation

The sample size was calculated based on an approximate population size of 155,739 in the study areas, using a 5 % margin of error, a 95 % confidence interval (CI), and a power of 80 % (1 - β). The estimated sample size was 385 participants, accounting for an anticipated response rate of 50 % and potential non-responders.

### 2.6 Data Analysis

Descriptive statistics, including frequencies and percentages, were reported for categorical variables. Continuous variables were summarised using means and standard deviations. Associations between variables were examined using chi-square tests for categorical data and *t*-tests for continuous data, as appropriate. Statistical significance was set at *p* < 0.05. Data were analysed using SPSS version 24.

**Ethical Approval:** The study met the institution's guidelines for protection of human subjects concerning safety and privacy. It was approved by the Research and Ethical Review Approval Committee (RERAC), Centre of Studies and Research, Ministry of Health, Oman, MOH/CSR/22/26382.

## 3 Results

A total of 503 participants were invited to participate, out of whom 467 agreed (response rate: 92.8 %). The main reason given for declining to participate was time constraints. The mean age of the participants was 32.5 years, with 32.8 % being male and 67.2 % female. This may be attributed to the higher proportion of females compared to males among visitors to primary healthcare centres(Table 1). The prevalence of OFDS usage was 76.9 % (359/467). Univariate analysis indicated significant associations between OFDS usage and factors such as younger age (*P* = 0.0), being single (P = 0.0), higher educational attainment (P = 0.0), not having any chronic diseases (P = 0.0), and physical inactivity (*P* = 0.0). Interestingly, no significant correlation was found between OFDS usage and obesity/overweight as determined by BMI measurements (*P* = 0.109).

**Table 1:** Comparison of baseline characteristics and associated risk factors among adults using versus not using online food delivery services in the Muscat region, Oman, January–June 2023 (*n* = 467).

Variables		Use of an online food delivery service		
		Yes n (%)	No n (%)	Chi-square test/ t-Testing
Age, (Years; Mean + SD)		31.1 (SD 8.1)	36.9 (SD 10.1)	⁎P = 0.0
Income (Omani Rials; Mean + SD)		804.2 (SD 494.0)	777.6 (SD 465.2)	⁎P = 0.7
Gender, n(%)	Male	111 (72.5)	42 (27.5)	P = 0.1
	Female	248 (79.0)	66 (21.0)	
Marital status, n(%)	Single	141 (85.5)	24 (14.5)	P = 0.0
	Married	201 (72.3)	77 (27.7)	
	Divorced	14 (73.7)	5 (26.3)	
	Widow	3 (60.0)	2 (40.0)	
BMI categories n (%)	Underweight	23 (85.2)	4 (14.8)	P = 0.1
	Normal	148 (81.3)	34 (18.7)	
	Overweight	113 (74.8)	38 (25.2)	
	Obese	73 (70.2)	31 (29.8)	
Educational level, n(%)	Primary School	2 (25.0)	6 (75.0)	P = 0.0
	Secondary School	95 (64.6)	52 (35.4)	
	Undergraduate	237 (84.9)	42 (15.1)	
	Postgraduate	25 (78.1)	7 (21.9)	
Employment, n(%)	Employed	218 (76.0)	69 (24.0)	*P* = 0.2
	Unemployed	81 (74.3)	28 (25.7)	
	Student	60 (84.5)	11 (15.5)	
Chronic diseases, n(%)	Yes	80 (69.0)	36 (31.0)	P = 0.0
	No	279 (79.5)	72 (20.5)	
Psychiatric illness, n(%)	Yes	3 (75.0)	1 (25.0)	P = 0.9
	No	356 (76.9)	107 (23.1)	
Medication use for chronic illness, n(%)	Yes	3 (75.0)	1 (25.0)	P = 0.9
	No	356 (76.9)	107 (23.1)	
Smoking, n(%)	Yes	10 (66.7)	5 (33.3)	*P* = 0.3
	No	349 (77.2)	103 (22.8)	
Exercise, n(%)	Yes	214 (73.5)	77 (26.5)	P = 0.0
	No	145 (82.4)	31 (17.6)	
Medication use for weight loss, n(%)	Yes	16 (76.2)	5 (23.8)	P = 0.9
	No	343 (76.9)	103 (23.1)	
Weight loss procedure, n(%)	Yes	16 (76.2)	5 (23.8)	P = 0.9
	No	343 (76.9)	103 (23.1)	

SD: Standard Deviation, BMI: Body Mass Index.

⁎ t-test.

Regarding the use of OFDS, a significant portion of participants rated their control over ordering food online as fair or good (49.3 % and 40.4 %, respectively). More than a third of participants cited living alone as the primary factor influencing their decision to order food online. Dinner was the most commonly ordered meal, preferred by nearly two-thirds of participants, followed by lunch and breakfast. The majority of participants also ordered desserts and snacks. On average, participants ordered food online approximately twice a week, spending an average of 11.14 Omani Rials (SD 12.8). Further details regarding the characteristics of participants utilising online food delivery services are provided in Table 2.

**Table 2:** Characteristics of adults using online food delivery services in the Muscat region, Oman, January–June 2023 (*n* = 359, 76.9 %).

Variables		N (%)
How do you describe your control when ordering food online?	No control [I order whatever and whenever to satisfy my cravings]	37(10.3)
	Fair control [I indulge myself sometimes, but not very often]	177(49.3)
	Good control [I rarely order and only when I really have to]	145(40.4)
What are the factors affecting your decision to order food online?	I live alone	132(36.8)
	I don't know how to cook + I work for long hours + I have no time + It is easy to order	67(18.7)
	I am lazy	66(18.4)
	I don't know how to cook	30(8.4)
	I have no time	19(5.3)
	I work long hours	16(4.5)
	I live alone and I am lazy	12(3.3)
	It is easy	8(2.2)
	I live alone + I don't know how to cook + It is easy to order + I am lazy	5(1.4)
	I have no time and it is easy to order	4(1.1)
Which meal do you order online the most?	Dinner	246(68.5)
	Lunch +Dinner	46(12.8)
	Lunch	31 (8.6)
	Breakfast	15(4.2)
	Breakfast +Dinner	13(3.6)
	Breakfast+Lunch+Dinner	6(1.7)
	Breakfast +Lunch	2(0.6)
What type of food do you usually order?	Desserts	246(68.5)
	Combination desserts, snacks and main dishes	67(18.7)
	Snacks	31(8.6)
	Main dishes	15(4.2)
What time do you usually order food online?	Night	246(68.5)
	Afternoon	32(8.9)
	Morning	13(3.6)
	Combination of the above times	68(18.9)
How many times per week do you order food using online delivery services? (Mean + SD)		2.13 (SD 1.49)
How much do you spend on ordering food online per week? (Omani Rials; Mean + SD)		11.14 (SD 12.84)

SD: Standard Deviation.

## 4 Discussion

The current study revealed a high prevalence rate of OFDS usage among Omani people. Younger age (*P* = 0.0), being single (P = 0.0), higher educational attainment (P = 0.0), not having any chronic diseases (P = 0.0), and physical inactivity (P = 0.0) were significantly associated with OFDS usage. Surprisingly, no significant association was noted between OFDS and obesity/overweight. Many participants felt they had fair to good control over online food ordering. Living alone was a significant factor influencing this choice, with dinner being the most ordered meal, and participants typically ordered food twice a week.

The high prevalence of OFDS usage is consistent with other studies in the literature (Almansour et al., 2020; Keeble et al., 2021; Stephens et al., 2020).The data suggests a remarkable increase in OFDS usage worldwide. The number of users had increased from 806.76 million in 2018 to 2128.69 million in 2024 (Statista, 2024a, Statista, 2024b).The number is even expected to increase to 2523.28 million in 2028 (Statista, 2024a, Statista, 2024b). Evidence indicates that a significant portion of the food ordered tends to be unhealthy, as demonstrated in the present study, with over two-thirds of participants frequently ordering desserts. A study conducted in three international cities – Chicago (United States of America), Amsterdam (The Netherlands), and Melbourne (Australia) – revealed that the majority of food options offered for delivery were not deemed healthy (Poelman et al., 2020). Likewise, a study conducted among adolescent participants in the United Arab Emirates found that the majority reported using OFDS applications on a weekly basis (65.4 %), with fast food being the preferred choice (85.7 % of users) (Saleh et al., 2024).

Understanding the reasons for OFDS use is essential for the development of future public health interventions. The current study indicates that living alone is the most frequently reported factor associated with online food delivery use. Most participants consider OFDS easily accessible and convenient for use, and this finding is consistent with other studies (Keeble et al., 2022; Dana et al., 2021).^14,15^ Another study showed that key factors influencing food selection included appearance and price (65.0 %), whereas taste and cost were the primary barriers to ordering healthy food (29.7 % and 28.2 %, respectively) (Saleh et al.,2024).

Younger people in the current study are more likely to use an OFDS, which is consistent with other studies (Dana et al., 2021; Buettner et al., 2023; Keeble et al., 2020; Algheshairy et al., 2022). A recent study in Saudi Arabia indicated that the majority of female Saudi users of food delivery applications were aged 18–24 years (64.9 %) Algheshairy et al.,2022). Moreover, younger people are often single, attaining a higher educational level (full-time student) and living away from family. The significant association with the aforementioned factors might be explained by the fact that a high percentage of younger people in the current study are studying in universities as full-time students and living away from their families, which may necessitate using a quick way of ordering food. In addition, many young adults residing alone often perceive themselves as lacking cooking skills, leading to diminished motivation to prepare their own meals (Andrade, 2020). The significant association between higher educational attainment and OFDS usage is consistent with the study conducted by Dana et al. (Dana et al.,2021) This might be similarly explained by the fact that many university students are living away from their families and therefore find the OFDS quicker and easier to use given their study commitments. Likewise, the fact that participants who are healthy, without a history of any chronic diseases, are more likely to use the OFDS might be attributed to the young age overall of the recruited participants. The research indicates that younger individuals are generally less attentive to their dietary habits (Wojcicki and Heyman, 2012), whereas those with chronic conditions tend to be more vigilant and mindful of their food choices (Lewis et al., 2009).

Surprisingly, with such a high prevalence rate of using an OFDS, one might expect a strong association with obesity and overweight, which the current study refuted. This contradicts other studies in the literature (Harahap et al., 2020; Mokhtazar and Hamirudin, 2020). The contradictory finding might be explained by a study conducted in Greece, which showed that the correlations between variables of the Theory of Planned Behaviour (TPB) and behaviours like healthy eating and exercise were stronger in the normal-weight group compared to the overweight/obese group (Psouni et al., 2016; Alyami and Alharbi, 2023) All in all, the majority of participants in the current study showed either fair or good control over online food ordering, which may have contributed to the non-significant association with BMI.

### 4.1 Limitations

The cross-sectional design of this study restricts the ability to establish causal relationships between OFDS usage and obesity, underscoring the need for future research employing prospective longitudinal designs to explore causality more robustly. The reliance on self-reported data for online food delivery service usage introduces the possibility of social desirability bias, as participants may underreport behaviours perceived as unhealthy. Similarly, recall bias may affect the accuracy of information provided, particularly regarding the frequency and types of meals ordered. These biases could lead to an underestimation of the relationship between OFDS usage and obesity.

The study's recruitment of participants exclusively from healthcare centres in the Muscat Governorate may limit the generalisability of findings to the broader Omani population. Although the Muscat population includes individuals from diverse regions of Oman, this urban-focused sampling may not fully capture behaviours and attitudes prevalent in rural areas or less urbanised regions. Furthermore, variations in socioeconomic factors, cultural influences, and access to OFDS across regions may result in differing patterns of use and associated health impacts.

Additionally, while this study identified a link between OFDS usage and obesity, this relationship requires further interpretation in light of contradictory evidence from other studies. For instance, some research suggests that the availability of healthier options on OFDS platforms could mitigate obesity risks, contrasting with findings indicating a preference for fast food. These nuances highlight the complexity of the OFDS-obesity relationship, suggesting that factors such as user demographics, platform design, and cultural attitudes toward food may play moderating roles. Finally, the broader implications of these findings, particularly their relevance to public health policy, warrant further exploration. This includes considering how technological advancements, such as app-based nutritional education and targeted interventions, could be leveraged to address obesity within the context of increasing OFDS usage. Future research should aim to address these gaps by incorporating diverse populations, longitudinal methodologies, and a comprehensive analysis of both individual and platform-level factors influencing dietary choices.

### 4.2 Recommendations

To promote healthier eating habits, targeted public health interventions should focus on nutrition and health education campaigns tailored to different demographic groups, emphasising the long-term health consequences of unhealthy food choices. Food delivery applications should be enhanced by integrating features such as detailed calorie and nutrient information for each meal, as well as personalised alerts or restrictions based on recommended daily caloric intake. Policymakers should collaborate with app developers to include tools that promote healthier meal options, such as filters for low-calorie or balanced meals and incentives for choosing healthier alternatives. Additionally, regular public health monitoring of fast-food consumption patterns should be established, incorporating a standardised questionnaire to capture comprehensive data on meal content, frequency, and preferences. This data could inform targeted interventions to reduce fast-food intake and its associated health risks. Lastly, while this study focused on the Muscat region, future research should include rural and urban areas across Oman to develop inclusive and region-specific health policies that cater to the diverse needs of the population.

## 5 Conclusions

The current study delineates a notable prevalence of OFDS utilisation among Omani individuals, with key associations identified with younger age, single status, higher educational attainment, absence of chronic diseases, and physical inactivity. While the widespread adoption of OFDS aligns with global trends, it raises concerns given the predominance of unhealthy food options. Understanding the drivers behind OFDS use, particularly among younger, educated individuals living independently, is crucial for informing future public health interventions. Although our findings challenge assumptions regarding the link between OFDS and obesity, further research employing prospective longitudinal designs is warranted to elucidate the causal relationships. Public health initiatives should prioritise education on healthy eating habits and advocate for improvements in food delivery app functionalities to promote better dietary choices.

## Funding

This research did not receive any specific grant from funding agencies.

## Ethical Approval

The Research and Ethical Review & Approval Committee (RERAC), Centre of Studies and Research, Ministry of Health, Oman, MOH/CSR/22/26382.

## CRediT authorship contribution statement

**Ahmed Yahya Al Kharusi:** Writing – review & editing, Writing – original draft, Methodology, Formal analysis, Data curation, Conceptualization. **Safa Khamis Ambusaidi:** Writing – review & editing, Writing – original draft, Resources, Methodology, Formal analysis, Conceptualization. **Marwa Abdullah Al Raisi:** Writing – review & editing, Writing – original draft, Resources, Methodology, Formal analysis, Conceptualization. **Haitham Mohammed Al Mahrouqi:** Writing – review & editing, Writing – original draft, Resources, Methodology, Formal analysis, Conceptualization. **Asma Ali Al Kendi:** Writing – review & editing, Writing – original draft, Resources, Methodology, Formal analysis, Conceptualization. **Muna Mohammed Almatrushi:** Writing – review & editing, Writing – original draft, Methodology, Formal analysis, Conceptualization. **Mohammed Juma Al Abdali:** Writing – review & editing, Writing – original draft, Resources, Methodology, Formal analysis, Conceptualization. **Sanjay Jaju:** Writing – review & editing, Writing – original draft, Validation, Formal analysis, Conceptualization. **Maisa Hamed Al Kiyumi:** Writing – review & editing, Writing – original draft, Validation, Supervision, Methodology, Formal analysis, Conceptualization.

## Declaration of competing interest

The authors declare that they have no known competing financial interests or personal relationships that could have appeared to influence the work reported in this paper.

## Data Availability

Data will be made available on request.
